# Toll-Like Receptors as Therapeutic Targets in Central Nervous System Tumors

**DOI:** 10.1155/2019/5286358

**Published:** 2019-05-21

**Authors:** D. M. Abarca-Merlin, C. Maldonado-Bernal, L. Alvarez-Arellano

**Affiliations:** ^1^Laboratorio de Neurociencias, Hospital Infantil de México Federico Gómez, Mexico City, Mexico; ^2^Facultad de Química, Universidad Nacional Autónoma de México, México City, Mexico; ^3^Laboratorio de Investigación en Inmunología y Proteómica, Hospital Infantil de México Federico Gómez, Mexico City, Mexico; ^4^CONACYT-Hospital Infantil de México Federico Gómez, México City, Mexico

## Abstract

In recent years, progress has been made in understanding the pathological, genetic, and molecular heterogeneity of central nervous system (CNS) tumors. However, improvements in risk classification, prognosis, and treatment have not been sufficient. Currently, great importance has been placed to the tumor microenvironment and the immune system, which are very important components that influence the establishment and development of tumors. Toll-like receptors (TLRs) are innate immunite system sensors of a wide variety of molecules, such as those associated with microorganisms and danger signals. TLRs are expressed on many cells, including immune cells and nonimmune cells such as neurons and cancer cells. In the tumor microenvironment, activation of TLRs plays dual antitumoral (dendritic cells, cytotoxic T cells, and natural killer cells activation) and protumoral effects (tumor cell proliferation, survival, and resistance to chemotherapy) and constitutes an area of opportunities and challenges in the development of new therapeutic strategies. Several clinical trials have been carried out, and others are currently in process; however, the results obtained to date have been contradictory and have not led to a definitive position about the use of TLR agonists in adjuvant therapy during the treatment of central nervous system (CNS) tumors. In this review, we focus on recent advances in TLR agonists as immunotherapies for treatment of CNS tumors.

## 1. Introduction

Central nervous system (CNS) tumors constitute a heterogeneous group of neoplasms that originate from many pluripotent and differentiated cell types, whose incidence and mortality are increasing worldwide. The GLOBOCAN 2018 database indicates that there were 296,851 new cases and 241,037 deaths from brain cancer compared with 257,000 and 189,000, respectively, in 2012 [1]. Factors that have been associated with increased risk of developing primary CNS tumors include hereditary syndromes and exposure to X-rays and gamma rays; however, in most cases, the etiology is unknown.

Several different types of tumors, both nonmalignant and malignant, have been identified in the CNS. The highest incidence of primary CNS tumors in adults is from meningiomas and neuroepithelial tumors (glioblastoma and pituitary tumors). Children and adolescents present with a higher incidence of embryonal tumors, mainly medulloblastoma, pilocytic astrocytoma, and ependymal tumors [1, 2].

Currently, conventional treatments, including surgical resection of tumors, craniospinal radiotherapy, and chemotherapy, are often successful. However, some CNS tumors are not amenable to surgical resection due to the depth of tumor infiltration or anatomical location of it. The side effects from treatments in patients significantly affect their neurological and psychological function and quality of life.

Advances have been made in understanding CNS tumor biology, but improvements in risk classification, prognosis, and treatment have not been sufficient. Recently, great importance has been placed on the tumor microenvironment, which is composed mainly of cancer cells, stromal cells, and immune cells [3]. The activation of the immune response is an important factor in the onset, development, and metastasis of cancer, and therfore, the immune system is a potential therapeutic target.

## 2. Toll-Like Receptors

The initiation of the innate immune response begins with the recognition of exogenous molecules from microorganisms, called microbial-associated molecular patterns (MAMPs), as well as the recognition of endogenous molecules considered to be danger signals called danger-associated molecular patterns (DAMPs). MAMPs and DAMPs are recognized by germline-coded receptors, called pattern recognition receptors (PRRs). This family of receptors consists of mannose binding lectin (MBL), Toll-like receptors (TLRs), nucleotide-binding oligomerization domains (NOD) receptors, dectin-1, NOD-like receptors (NLRs), mannose receptors, and pentraxins, among others. Principal effector functions of PRRs include the activation of the transcription of genes involved in the immune response (cytokines, chemokines, growth factors, adhesion molecules, and costimulation), opsonization, phagocytosis, activation of the complement system, proliferation, and cell death [4, 5].

TLRs are type I transmembrane proteins, characterized by an extracellular domain with leucine-rich repeats (LRR) motifs. Through this domain, TLRs recognize structures present in certain groups of pathogens and endogenous molecules released in situations that result from physiological stress. Additionally, TLRs possess an intracellular domain, called TIR (Toll/IL-1R) similar to the interleukin-1 receptor (IL-1R) family, which leads to the activation of a signaling pathway [6]. The Toll protein was identified for the first time in* Drosophila melanogaster* as a fundamental receptor for dorsoventral polarity during the early phases of embryonic development of the fly [7]. Subsequent studies have shown that the Toll protein has a very important function in the immune system of the adult insect, mainly during infections by bacteria and fungi. Currently, it is known that TLRs are evolutionarily conserved from invertebrate organisms such as* Caenorhabditis elegans* to mammals [8]. To date, 13 members of this family have been found in mammals, including 10 in humans (TLR1 to TLR10) and one pseudogene [9]. The expression of TLRs is not limited to the cell immune system as they are expressed in other cell types. In the central nervous system, TLRs are present in glial cells (microglia, astrocytes, and oligodendrocytes), neuronal progenitors, mature neurons, and cancer cells (Table 1).

TLR ligands include MAMPs, endogenous molecules, and synthetic agonists. For example, TLR2, in conjunction with TLR1 or TLR6, recognizes a wide variety of MAMPs and DAMPs, such as lipoproteins, peptidoglycans, lipoteichoic acid, and zymosan. TLR3 recognizes double-stranded RNA (dsRNA) and polyinosinic:polycytidylic acid [poly(I:C)]. TLR4 recognizes lipopolysaccharide (LPS) and TLR5 flagellin [10]. TLR7 recognizes single-stranded RNA (ssRNA), microRNAs, small interfering RNAs (siRNA), and imidazoquinoline derivatives such as imiquimod (IMQ) and resiquimod (R848) and guanine analogs such as loxoribine. TLR8 is phylogenetically similar to TLR7 and preferentially recognizes bacterial RNA, ssRNA from viruses, and synthetic agonists such as R848. Finally, TLR9 is known to recognize cytosine-guanine motifs bound by nonmethylated phosphodiester (CpG) bonds and synthetic CpG oligonucleotides (ODN) and immunoglobulin-DNA complexes [5, 9, 10]. Activation of TLRs induces the recruitment of adapter proteins (MyD88, TRIF, etc.) that bind the TIR domain triggering a signaling cascade and activating transcription factors such as nuclear factor kappa B (NF-*κ*B), activator protein-1 (AP-1), and interferon regulatory factors (IRFs) [11, 12], which in turn trigger transcription of genes that participate in the immune response and cellular processes such as proliferation, migration, and cell death [11–17].

## 3. TLRs and Immune Responses to Tumors

In the tumor microenvironment, TLR signaling can induce anti- or protumor effects which depends on the cancer subtype and the cells of the immune system that infiltrate the tumor [18]. There is great controversy for some TLRs which are explained by the tumor models used in the experiments. It has been documented that stimulation of TLRs may have antitumor effects through an intermediary immune cells response or directly on tumor cells, which improves the antitumor immune response and leads to apoptosis of tumor cells [19, 20]. Apoptosis of tumor cells can be generated by different mechanisms downstream of TLR3 activation in different cancer cell lines, as indicated by lower survivin expression and negative regulation of XIAP, FLIP, Bcl-xL, and Bcl-2 have been observed [21–23] and by a large number of cells positive for proapoptotic caspase-8 and caspase-3 [21].

The immune system has been shown to be more efficient activating the response to MAMPs than recognizing and eliminating tumor cells. Pharmacological studies have shown that the activation of signaling pathways initiated by TLRs through recognition of MAMPs and DAMPs, but not tumors, induces the production of mediators such as type I interferons (IFN), which can be used therapeutically to modify immunotolerance and produce antitumor effects.

The antitumor immune response depends largely on the cells presenting professional antigen-presenting cells, such as dendritic cells (DCs) [24, 25]. DCs express all TLRs and exert effects on T and B lymphocytes; they are the bridge between innate and adaptive immune responses. IFNs are necessary for an efficient immune response to tumors [26]. Therefore, activation of the TLR-IFN type I signaling pathway is of therapeutic importance in that it eliminates DC-induce tolerance and generates an antitumor response. Additionally, DCs activated by TLRs can mediate antitumor responses, by the presenting antigens, thereby initiating a T cell response, and by inducing cytotoxicity in tumor cells [27]. It has been documented that DCs activated by TLR7 ligands can induce antitumor responses by cell lysis [28]. On the other hand, the activation of TLR5 with flagellin can increase DC antitumor activity [29]. The death of tumor cells mediated by DC generates a more efficient antigen presentation for cytotoxic T lymphocytes, amplifying the antitumor response. DCs stimulated with TLR9 also produce antitumor responses [30].

IFN has the ability to regulate the functions of natural killer cells (NK) and is very important for the modulation of tumor growth. A murine model of melanoma demonstrated that myeloid DCs can be activated via TLR3 with poly(I:C), inducing NK cell response and regression of tumors. It is now known that the NK-activating molecule dependens on IRF-3 to create a link between myeloid DCs and NK cells [31].

In addition, regulatory T cells (Tregs) (CD4+CD25+FoxP3+) have a very determinant role in the immune response, and to induce tolerance; it is known that the TLRs of DCs regulate the activation of Tregs through signals that block the immunosuppression by IL-6 [32]. Also, TLR8 agonists can inhibit the function of Tregs independently of DCs and promote an antitumor response [33].

The poly(I:C) agonist of TLR3 can cause tumor regression, such that tumor macrophages are transformed into tumor suppressor macrophages that produce inflammatory cytokines (M1 macrophages). This change is mediated by TNF-*α* through an independent pathway of MyD88 [34]. Furthermore, TLR9 agonists can also exert antitumor effects by suppressing angiogenesis [35]. TLR-induced interferon has an important role, because it reduces tumor growth blocking angiogenesis and metastasis [36, 37]. However, tumor cells may also activate TLRs and, coupled with a specific type of tumor, can cause death, survival, or proliferation of tumor cells, including resistance to chemotherapy [18].

## 4. Immunotherapy Targeted to the Activation of TLRs

TLR agonists have been considered therapeutic targets for treating different cancers [38]. Many synthetic ligands are being investigated for use in immunotherapy. ODNs are the most commonly used TLR agonists in therapy; they are potent activators of both innate and adaptive immununity and thus are capable of inducing cytokine production and activating NKs, dendritic cells, monocytes, and antitumor T cell responses [39]. Stimulation of TLR9 with ODN 1826 induces caspase-3-dependent apoptosis in gliomas and prolongs the survival of C57BL/6 mice with an intracranially implanted glioma cell line (GL261). Moreover, mice treated with ODN 2138 showed no evidence of enhanced survival [40]. In addition, ODNs enhance survival and prime long-term immunity in mice with two separate glioblastoma tumors in wich only one was treated. This may suggest that treatment with CpG-ODN could be effective in tumor cells located at some distance from the application site [41]. The antitumoral effect was not mediated by direct toxicity but instead involved cells of the immune system, including NK cells, macrophages, microglial cells, and CD8 T cells [41].

In another study, Grauer* et al*. showed that there are low to undetectable levels of TLR5, TLR7, and TLR9 in GL261 cells; surprisingly, in C57BL/6 mice, a single intratumoral injection of CpG ODN 1668 inhibited glioma growth and cell proliferation in a cell-type specific manner. CpG ODN 1668 was superior in the elimination of murine gliomas (median survival >90 days) when compared with PAM_3_Cys-SK4 (median survival= 34.5 days), while LPS and poly(I:C) did not show a significant effect on tumor growth (median survival = 27 days). Similar to ODN 1668, R848 also extended the survival of glioma-bearing mice but not as effectively (median survival >36.5 days) [42].

Subcutaneous vaccination of CpG-ODN 2006 with glioma cell lysate (cell line GL261) in glioma-bearing mice had a potent antitumoral effect with a cure rate of 55%; the mice showed a significant increase in activated DCs and a considerable expansion of T lymphocytes, which produced IFN-*γ* and lysed glioma cells. These data support the idea that priming T cells extracranially with CpG-activated DCs with tumor antigens is better than administering intratumoral CpG ODN [41, 43]. According to the authors, this method is more effective and simple, and potentially safer for the administration of CpG ODN in glioma immunotherapy. However, it is known that cell line gliomas are more immunogenic than arising human gliomas; therefore, more studies are needed [44].

The CpG ODN effect is enhanced by using a vehicle that promotes internalization to target cells; carbon nanotubes (CNTs) have been tested as a thiolated CpG (sCpG) delivery vehicle into the tumor-associated inflammatory cells in a murine glioma model. CNT-sCpG delayed tumor growth, and 50-60% of mice with established gliomas were cured. This antitumoral effect was accompanied by a sustained elevation of NK cells in the circulation and macrophage infiltration into the brain. sCpG alone enhanced mouse survival, but the effect was less than when mice were treated with CNT-sCpG [45].

Another advantage of using of TLR agonists in immunotherapy is that TLR activation may also have a systemic effect. Xiong* et al*. demonstrated that topical administration of IMQ significantly increased the number of DCs and tumor-reactive T cells that reached the glioma site. Additionally, soluble IMQ inhibited the proliferation of GL261 cells in a TLR7-independent manner because TLR7 mRNA was not expressed in the tumor cells [42, 46]. The inhibitory effects of IMQ in glioma cells do not require TLR7 expression, and the mechanism by which IMQ inhibits tumor growth could be due to the adenosine receptor-mediated signaling pathway [46]. Similarly, another study found that TLR7/TLR8 is not expressed in the glioma rat model CNS-1; however, the activation of TLR7/8 by R848 alone was sufficient to cause rejection of the smaller established glioma in CNS-1 [47]. LPS injected intratumorally in a glioblastoma model induced near-complete subcutaneous tumor elimination in wild-type BALB/c mice and a 50% reduction in TLR4 knockout mice. However, it did not confer a substantial benefit in intracranial glioblastoma-bearing mice. Analysis showed no TLR4 expression in the tumors taken from wild-type mice. However, a neutrophilic and macrophage-rich infiltrates were found in both tumors. The evidence indicates that the immunity-related antitumoral effect of LPS is not completely mediated by TLR4 [48]. Together, these findings suggest the participation of the immunological and stromal components of the tumor microenvironment.

The use of LPS in therapy against CNS tumors requires careful study; several reports have found a neurodegenerative and inflammatory effect of this TLR4 agonist and hence have suggested less toxic alternatives [49, 50]. Kawanishi et al. reported that Spirulina complex polysaccharides (CPS) initiated an antitumoral response against glioma and induced a greater production of IL-17 than LPS in C3H/HeJ mice; however, this result is opposite that for C3H/HeN mice. The results confirm that these effects are dependent on TLR4 signaling. The anti-IL-17 antibodies inhibited the growth of glioma cells in both mouse strains (C3J/HeN and C3J/HeJ) but did not increase the growth suppression by Spirulina CPS in C3J/HeN mice. In addition, C3H/HeN mice treated with CPS had lower concentrations of IL-17, developed acquired immunity, and expressed low levels of CD31 (angiogenesis marker). Finally, T cells, macrophages, and NK cells were identified as being responsible for glioma growth suppression through Spirulina CPS-TLR4 signaling. The authors concluded that the antitumoral effect of CPS is due to angiogenesis suppression and in part to the ability to regulate IL-17. They also demonstrated that the antitumoral effect of* E. coli* LPS is induced by IL-17 and IFN-*γ* production, but LPS had no effect on glioma angiogenesis. In contrast, other studies showed that Spirulina CPS could cause NF-*κ*B induction via TLR2 and TLR4. These findings may suggest that TLR4 is not the only path for Spirulina CPS to induce its effect [51]. Another study showed that the absence of TLR4 inhibited the growth of U-87 tumor xenografts. Furthermore, TLR4 gene deficiency induced apoptosis process (caspase-3-dependent), resulting in a decrease in tumor growth. This suggests that TLR4 is a biomarker of interest for tumor metastasis and prognosis [52].

Nevertheless, the antitumor effect of LPS has been shown. Hua et al. reported that LPS-TLR4 activation fosters glioma growth and decreases mouse survival; however, it did not promote proliferation in vitro. This activation also downregulated in a dose-dependent manner glial fibrillary acidic protein (GFAP). LPS treatment produced slight phosphorylation of MAPKs, ERK, JNK, and p38 but significantly increased phosphorylation of NF-*κ*B and activation of the MyD88-dependent Notch pathway. Notch inhibition reversed the downregulation of GFAP, suggesting that LPS reverses glioma differentiation via the MyD88-dependent Notch pathway [53].

Despite these results, the participation of TLR2 in the antitumor responses in the CNS has been controversial. It was reported that TLR2 activation with a synthetic bacterial lipoprotein administered jointly with tumor antigen-specific CD8 T cells increased long-term survival and immune memory in a murine glioma model GL261 [54]. However, the protumorigenic function of TLR2 has also been demonstrated. In murine GL261 glioma cells implanted in TLR2 knockout mice, the lack of TLR2 resulted in significantly smaller tumors, reduced membrane type 1 matrix metalloprotease (MT1-MMP) expression, and enhanced survival rates compared with wild-type control mice. Agonists of TLR2 (Pam_3_CSK_4_ and MALP2) induce the upregulation of MT1-MMP expression, promoting glioma expansion and progression [55]. Nevertheless, a distinctive dysfunction in TLR2 ligand-induced tyrosine phosphorylation of STAT1 has been observed in malignant cells but not normal glia. Microglia, astrocytes, and neuroblastoma cells treated with LTA and Pam3CSK4, two TLR2 ligands, induced tyrosine phosphorylation of STAT1 in both astrocytes and microglia, but it was not detected in neuroblastoma or different glioma cell lines (GL26, U87, and U373) [56].

In addition, the possible therapeutic potential of some endogenous ligands (e.g., DAMPs) has been demonstrated. Curtin et al. developed immunotherapy using adenoviral vectors expressing Fms-like tyrosine kinase 3 ligand (Flt3L) and thymidine kinase (TK) administration into glioblastoma. While the Flt3L induces DC infiltration into the brain parenchyma, TK is a conditional cytotoxic gene. Later, researchers identified an endogenous TLR2 agonist called high-mobility-group box 1 (HMGB1), wich is released by dying tumor cells as a result of tumor cell killing. When HMGB1 was blocked, Flt3L/TK-induced glioma brain tumor regression was inhibited. Tumor-derived HMGB1 triggers a CD8+ T cell antiglioblastoma response and induces TLR2 signaling [57]. Nevertheless, HMGB1 is not a specific ligand for TLR2; it can also be recognized by TLR4, TLR9, and RAGE and activate multiple signaling pathways (NF-*κ*B, ERK1/2, p38, and STAT3) and subsequently the regulation of cytokines, chemokines, adhesion molecules, cell proliferation, survival, differentiation, migration, phagocytosis, autophagy, and tumorigenesis [58–60]. These findings taken together demonstrate that TLR signaling in CNS tumors is highly heterogeneous as is the resulting response. Furthermore, there is evidence NF-kB can be activated independently of TLRs. Tumorigenesis has been associated with the activation of NF-kB in glioblastoma multiforme [61]. However, in glioma cell lines (A172 and LN229), TNF*α*-induced NF-*κ*B activation is partially dependent on TLR4 and involves both MyD88 and TRIF [62].

Several clinical-phase studies have been carried out, and others are currently in process; however, the results obtained so far are controversial and have not led to a definitive position about the use of TLR agonists as adjuvant therapy to treating tumors of the CNS.

Phase I clinical studies have been conducted to establish the safety profile of CpG-28 in patients with recurrent glioblastoma. Patients were treated with increasing doses of CpG-28 and evaluated for at least four months. Two patients showed tumor reduction of 29% and 20% in the largest perpendicular diameters associated with reduced mass effect and decreased surrounding edema. Two other patients had a stable disease for more than four months. At the time of the antitumor response analysis, 20% of patients had died (n=24), and 28% experienced one-year survival; the median survival was 7.2 months. In conclusion, phase I trials and preclinical models demonstrated that local administration of CpG ODN in glioblastoma-bearing patients and those with recurrent glioblastoma is possible and tolerated at doses up to 20 mg [43]. Therefore, Ursu* et al*. conducted a phase I trial with patients with different types of cancer, including ependymoma (n=1), glioma (n=1), oligodendroglioma (n=1), oligoastrocytoma (n=1), and glioblastoma (n=15), and CpG-28 was administered to each patient. In some cases, patients received CpG treatment alone or in concomitantly with oncological treatment. The results showed heterogeneity among patients (n=29). Apparently, there was no significant survival between the groups treated with CpG-28 alone or CpG/oncological therapy. However, three patients showed remarkable changes. The patient with grade III ependymoma was stable during the protocol and remained alive 6 years after the study. The patients with grade III anaplastic oligoastrocytoma and glioblastoma showed clinical improvement after treatment with CpG28/bevacizumab, remained stable, and died at 12.5 months and 8.8 months, respectively [63].

**Figure 1:**
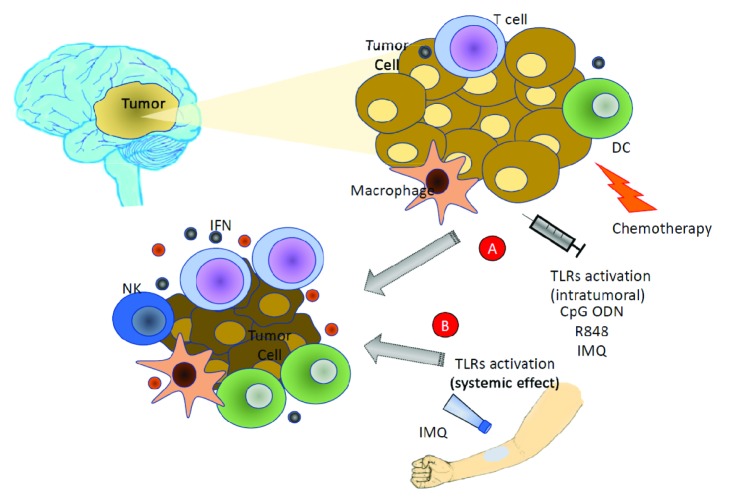
Summary mechanisms of TLR agonists as immunotherapy for CNS tumors. Activation of TLRs induces cytokine production, active NKs, dendritic cells, macrophage, T cell, and tumor cell apoptosis. TLR agonists can have local effect (A) and can also have a systemic effect (B).

In another phase I study, vaccination with autologous DC pulsed with glioma tumor lysate used as an adjuvant following surgical resection with standard chemoradiotherapy was determined to be safe, as it did not induce dose-limiting toxicities. In addition, the study authors used “boost” vaccinations with innate immune response modifiers (TLR agonists), 5% imiquimod, or poly-ICLC because these agonists may promote DC activation and priming of T cells; these vaccinations did not have any additional toxicity or adverse events. Interestingly, the median survival of patients treated with the vaccine was 31.4 months, compared to glioblastoma patients who had resection and were treated with concomitant chemoradiotherapy, where the median survival was 18.6 months [64] (Figure 1).

A phase II trial was performed to evaluate the efficacy and tolerance of a CpG ODN (10 mg/mL) treatment in recurrent-glioblastoma subjects. The authors did not find any progression-free survival in any of the patients evaluated, but the study had some long-term survivors, suggesting that some individuals might benefit from this treatment. Other studies need to be done with more patients to confirm whether side effects were caused by the CpG ODN treatment and to clarify which subgroup of patients benefit from the treatment [65]. Additional trials should be carried out with a greater number of patients to clarify the effect of immunotherapy targeting the activation of TLRs in tumors of the CNS.

## 5. Conclusion

The response induced by the activation of the TLRs leads to protumor or antitumor effects. Factors that determine the type of response include the agonists employed, type of cancer, the expression levels of TLRs, and the tumor microenvironment.

The molecular mechanisms through which TLRs modulate initiation, development, and tumor progression are not fully understood, but evidence shows their participation in processes such as apoptosis, angiogenesis, and proliferation of tumor cells. Clinical studies have shown the relevance and therapeutic potential of using TLR agonists in the treatment of tumors of the CNS. Future studies should be aimed at understanding the immunobiology of different malignancies originating in the CNS and establishing the efficacious and safety of immunotherapy based on the activation of TLRs that leads to establishing therapeutic alternatives for the treatment of cancer.

## Figures and Tables

**Table 1:** TLR expression in normal and neoplastic CNS cells.

Receptor	Cell/Tissue	mRNA/Protein	Specie	References

TLR1	Microglia, neurons	+/+	human, mouse	[66–68]
	Astrocytes	+/nd	human, mouse	[40]
	Glioma ∗(GL261, U251, U87, SF126)	+/-	human, mouse	[40, 53]
	Astrocytoma, Glioblastoma ∗(U87MG, A172)	+/+	human	[69]

TLR2	Oligodendrocytes	+/nd	human	[66]
	Microglia, astrocytes, neurons	+/+	human, mouse	[66–68, 70–74]
	Glioma ∗(GL261, U251, U87, SF126)	+/-	human, mouse	[40, 53]
	Medulloblastoma	nd/+	human	[75]
	Astrocytoma, Glioblastoma ∗(U87MG, A172)	+/+	human	[69]

TLR3	Astrocytes	+ /-	human, mouse	[66, 70]
	Oligodendrocytes, microglia, neurons	+/+	human	[66–68, 70, 72, 73, 76]
	Glioma ∗(GL261, SF126)	+/+	human, mouse	[40, 53]
	Medulloblastoma	nd/+	human	[75]

TLR4	Microglia, neurons	+/+	human	[66–68, 72]
	Astrocytes	+/+	human, mouse	[40, 71, 72]
	Glioma ∗(GL261, U251, U87, SF126)	+/-	human, mouse	[40, 53]
	Astrocytoma, Glioblastoma ∗(U87MG, A172, U118, LN229)	+/+	human	[62, 69, 77]

TLR5	Microglia	nd/+	human	[66]
	Astrocytes	+/+	human, mouse	[40, 71, 72]
	Glioma ∗(GL261, U251, U87)	+/-	human, mouse	[40]
	Astrocytoma, Glioblastoma ∗(U87MG, A172)	+/+	human	[69]

TLR6	Microglia, microglia ∗(EOC13)	+/+	human, mouse	[66, 67, 78]
	Neurons	+/+	mouse	[72]
	Astrocytes	+/nd	human, mouse	[40]
	Glioma ∗(U251; SF126)	+/-	human	[40, 53]
	Astrocytoma, Glioblastoma ∗(U87MG, A172)	+/+	human	[69]

TLR7	Microglia	+/nd	human	[66, 67]
	Neurons	+/+	human	[76]
	Astrocytes	+/nd	human, mouse	[40, 79]
	Glioma ∗(GL261, U251, U87)	+/-	human, mouse	[40]

TLR8	Microglia	+/nd	human	[66, 67]
	Neurons	+/+	human, mouse	[66, 72, 76]
	Astrocytes	+/nd	human, mouse	[40, 67]
	Glioma ∗(GL261, U251, U87)	+/-	human, mouse	[40]

TLR9	Neurons (differentiated from ∗SH-SY5Y)	nd/+	human	[72, 80]
	Astrocytes	+/nd	human, mouse	[40, 71, 72]
	Glioma ∗(GL261, U251, U87, SF126)	+/+	human, mouse	[40, 53, 77]

TLR10	Microglia	nd/+	mouse	[66]
	Glioblastoma ∗(T387, T3832, T4121)	+/+	human	[81]

TLR11	Microglia	nd/+	mouse	[66]
	Brain	+/-	mouse	[82]

TLR12	Brain	+/-	mouse	[82]

TLR13	Brain	+/-	mouse	[82]

+, positive expression; -, negative expression; nd, not determined expression; ∗cell lines.
